# Data sharing as the foundation of discovery: ADNI and breakthroughs in Alzheimer's disease

**DOI:** 10.1002/alz.14129

**Published:** 2024-07-30

**Authors:** Alessio Travaglia, Steve Hoffmann

**Affiliations:** ^1^ Translational Science, Neuroscience Foundation for the National Institutes of Health North Bethesda Maryland USA; ^2^ Science Partnerships, Translational Science Foundation for the National Institutes of Health North Bethesda Maryland USA

**Keywords:** ADNI, Alzheimer's disease, biomarkers, dementia, FNIH, innovation, neuroimaging, open data, public‐private partnerships

## Abstract

The COVID pandemic has shown that when the research community comes together, we can conquer the most complex biomedical challenges. Collaboration and teamwork among federal agencies, private organizations, and researchers have been crucial in the development of vaccines and therapeutics against COVID.

Possibly the first example of such cross‐functional collaboration is the Alzheimer's Disease Neuroimaging Initiative (ADNI), the largest and longest continually monitored Alzheimer's study. ADNI was designed and operated as a public‐private partnership, managed by the Foundation for the National Institutes of Health.

This article shows how recent successes in the Alzheimer's field are directly a result of ADNI's open and transparent sharing of knowledge, expertise, and resources, which have allowed researchers to advance their understanding of Alzheimer's and tackle challenges in a relatively short period of time. ADNI's approach to open‐source innovation also served as a model for addressing other complex diseases and led to numerous collaborative research initiatives.

**Highlights:**

- The Alzheimer's Disease Neuroimaging Initiative (ADNI) was designed, structured, and operated as a public‐private partnership, managed by the Foundation for the National Institutes of Health.
- The recent successes in the Alzheimer's field are directly a result of ADNI's efforts.
- Open and transparent sharing of knowledge, expertise, and resources allowed researchers to advance their understanding of Alzheimer's and tackle challenges in a relatively short period of time.

The COVID‐19 pandemic demonstrated that when stakeholders across the research community come together, we can conquer even the most complex biomedical challenges. Collaboration and teamwork among federal agencies, private organizations, and researchers have been crucial in the development of vaccines and therapeutics against COVID‐19. By sharing knowledge, expertise, and resources, researchers can expand their thinking and add new knowledge to complex problems, enabling them to tackle challenges in a relatively short period of time.

Possibly the first example of such a cross‐functional, open‐source collaboration is the Alzheimer's Disease Neuroimaging Initiative (ADNI), the largest and longest continually monitored Alzheimer's disease (AD) study. ADNI was launched in 2004 when the National Institute on Aging (NIA) at the National Institutes of Health (NIH) recognized the critical need to develop and validate biomarkers to improve the speed and success rate of clinical trials for AD.^1^

Understanding that such a colossal undertaking required significant investment, intellectual contribution, and a variety of skill sets, ADNI was designed, structured, and operated as a public‐private partnership, managed by the Foundation for the National Institutes of Health (FNIH) for nearly two decades.

ADNI was the first large collaboration among government organizations such as the NIH, the Food and Drug Administration (FDA), pharmaceutical organizations, and nonprofit organizations. This was a revolutionary concept at the time: It was unimaginable for researchers from different institutions, private and public organizations, to work together efficiently. ADNI was further envisioned as an *open* partnership—allowing a variety of stakeholders to explore fundamental questions that no individual organization could have accomplished on its own.

While recent FDA approvals of the first disease‐modifying therapies for Alzheimer's have received much fanfare, the open dataset of foundational research from ADNI that drove those discoveries has received less attention. Recent successes in the Alzheimer's field are directly a result of ADNI's efforts in open and transparent sharing of knowledge, expertise, and resources, which allowed researchers to expand their thinking and add new knowledge to complex problems, enabling them to tackle challenges in a relatively short period of time.

## 1 BACKGROUND

At the outset of ADNI, a major challenge posed by AD was the difficulty in precise and accurate diagnosis. At the time, diagnosis of AD was almost exclusively based on a physician's assessment, as there was minimal knowledge of molecular biomarkers that distinguish AD from other forms of dementia.

The pharmaceutical industry was developing disease‐modifying treatments to be tested, but enrolling appropriate patients proved an unexpected obstacle. For example, an after‐the‐fact analysis of a failed phase 3 pivotal study of a promising therapeutic showed that more than one in five patients enrolled in the study had non‐Alzheimer's dementia.^2^

Furthermore, clinical trials in AD were limited mainly to measurements in the form of questionnaires that assess cognition and functioning, like the ability to complete “activities of daily living.” Function and cognition, clearly, are extremely important, but at the same time these measurements are not sufficiently precise to detect the effects of treatments to slow AD progression.

Magnetic resonance imaging (MRI) and positron emission tomography (PET) biomarkers offered more precise alternatives to cognitive tests to assess disease progression—and ADNI was established primarily to fill this need. To achieve this, ADNI enrolled a large cohort (>800) of participants across the spectrum of the disease and developed standardized methods for taking clinical, cognitive, MRI, PET, biofluid, and genetics measurements.^3^ One aim was to develop biomarkers that could consistently identify the disease with high sensitivity and specificity at an earlier stage and to better monitor disease progression and treatment effects.

Prior to the funding of ADNI, the NIA realized that the amount of MRI and PET data that ADNI could generate would be enormous, so the NIA held discussions about how the data would be shared. There was to be no embargo on the availability of the resources generated by ADNI—no special access or period of exclusive use with respect to data—for either the ADNI investigators or the private partners. Additionally, the decision was made that there would be no preemptive intellectual property rights associated with the data in the database. The data and the samples would be open to the entire scientific research community, establishing a precedent for current discussions and policies that facilitate the transparency of federally funded research.

As a result, ADNI data have been queried millions of times by investigators around the world, from government, academic, and company research sites. As of 2023, there have been over 7 million downloads of ADNI data worldwide, and over 4400 publications using ADNI data.^4^ Applications for ADNI data have been from university researchers, followed by pharmaceutical/biotechnology companies, scanner manufacturers, government scientists, and other members of the public, including high school teachers.

### 1.1 The role of the ADNI Private Partners Scientific Board

Since inception, ADNI has undergone multiple peer‐reviewed renewals and has received continuous federal funding support over multiple phases. In addition, the FNIH managed more than 160 private partners and individual donors, who contributed $65 million in addition to the $145 million from the public sector, totaling a remarkable $210 million.

While the substantial financial investment was indeed pivotal to ADNI's success, it is imperative to acknowledge the invaluable guidance and support provided through the Private Partners Scientific Board (PPSB).^5^ The PPSB was established as an independent advisory board comprising members from the private sector, including biotechnology, diagnostics, and nonprofit organizations. Their contributions extended beyond mere financial support; they offered critical insights, advice, and suggestions that were instrumental in advancing ADNI's mission.

The various workgroups—Clinical Endpoints, PET Imaging Endpoints, MRI Core Assessment and Privacy, Fluid Biomarkers, Genetics, and Systems—played a crucial role in evaluating emerging technologies and shaping ADNI's structure and focus to optimize the treatment, monitoring, and care of AD patients. In addition to providing expertise and a private‐sector perspective, the PPSB also financed and executed supplementary projects beyond the original scope of ADNI.

Endpoints now routinely utilized in clinical trials, such as fluorodeoxyglucose (FDG) PET, amyloid PET, and the Alzheimer's Disease Assessment Scale–Cognitive Subscale (ADAS‐Cog), owe their refinement, enhancement, and widespread adoption to the diligent efforts and guidance of the PPSB workgroups.

The current phase of ADNI (ADNI4) continues the program's legacy of developing tools and methodologies to advance treatment trials for Alzheimer's and related dementias. Although ADNI4 is supported solely by a substantial increase in federal funding, the PPSB, now managed by the Alzheimer's Association, maintains its role of ensuring sustained collaboration and interaction between the public and private sectors.

### 1.2 Impact and open science

When ADNI was launched, the scientific understanding of the brain and neurological disorders was half a century behind our understanding of other tissues and disorders. ADNI's approach to open data sharing fundamentally changed the way we understand how the brain functions and how AD progresses.

Discoveries made through ADNI's open dataset of biosamples have shaped the way research is done, from designing drugs and biomarker‐directed therapies, to screening and selecting patients for clinical trials, to measuring whether a drug is working or not and ultimately whether a drug and trial are successful or not. Importantly, none of the recently approved Alzheimer's therapeutics would have been possible without the open collaboration fostered by ADNI.

The unfettered analysis of data generated by ADNI has ushered in a golden era of neuroscience. Biomarkers are an increasingly critical foundation for studying neurological disease; before ADNI, the scientific capability to measure, detect, or discern important biomarkers was very limited.

This change in viewpoint altered the way AD clinical trials were designed and conducted, with a shift toward identifying and treating patients with pre‐ and early symptomatic AD, which is also reflected in the NIA/Alzheimer's Association Workgroup's diagnostic guidelines for AD.^6^

In addition to disease staging, subsequent trials of disease‐modifying therapies require biomarker screening as an inclusion criterion to prevent the enrollment of individuals with non‐Alzheimer's dementia.^7^, ^8^, ^9^

### 1.3 ADNI's role in fostering public‐private partnerships and data sharing for neuroscience research

The lessons learned from ADNI have catalyzed numerous projects and ongoing research collaborations between the public and private sectors, exemplified by initiatives such as the Accelerating Medicines Partnership (AMP) Program for Alzheimer's Disease (AMP AD) and the FNIH Biomarkers Consortium.

AMP is a public‐private partnership program aimed at accelerating the development of new medicines and enhancing the understanding of disease biology. Managed by the FNIH and launched in 2014, AMP represents a collaboration between the NIH, FDA, multiple biopharmaceutical companies, and non‐profit organizations. One of the key initiatives within AMP is the AD program, which seeks to identify and validate new drug targets and biomarkers for the early detection and treatment of AD. This collaborative approach leverages the expertise and resources of multiple stakeholders to expedite the discovery of new therapies.

The Biomarkers Consortium unites various partners to identify, develop, and qualify potential biomarkers to enhance drug development and regulatory decision making. Through multiple Biomarkers Consortium projects, we are now at a critical juncture in developing a scalable blood biomarker for several neurodegenerative diseases. Specifically, the top‐performing amyloid‐beta (Aβ) and phosphorylated tau (pTau) assays are being evaluated and compared as predictors of amyloid positivity in AD.

ADNI's success has also paved the way for numerous other public‐private partnerships in neuroscience, demonstrating the substantial benefits of collaboration between academia, industry, and government. Inspired by ADNI's achievements, other groups have initiated similar partnerships with the goal of accelerating the discovery of treatments for various neurological disorders.

## 2 CONCLUSION

ADNI's approach to open‐source innovation catapulted our understanding of the way the brain functions and how AD progresses.

When ADNI was established, the concept that data generated by the initiative would be shared openly and without embargo to all qualified researchers worldwide was a relatively new and radical one. Research data even now are often considered to be owned by investigators, who guard it from competitors.

Conversely, the sharing of all ADNI data has benefited the entire pharmaceutical industry, from the development of standardized biomarkers to the use of imaging in precise clinical trial enrollment. Now that multiple treatments have emerged that demonstrate the possibility to slow the progression of AD via a direct and measurable impact on a disease biomarker, the many accomplishments of ADNI have served as a model for other initiatives and programs.^10^

There is a new territory to explore in the underlying biology of Alzheimer's. For example, Alzheimer's may be “more than one disease”—and multiple aspects of an individual's biosignature will predict responsiveness to current treatments and offer clues for the next generation of therapeutic innovation.

We also know Alzheimer's affects people differently. Race, wealth, education, fitness, along with many other factors influence the course of AD.^11^, ^12^ Developing personalized medicines increasingly needs to confront these difficult topics with open and transparent sharing of data and analysis.

This complexity is why open‐source innovation remains critical to continued progress against AD and other neurodegenerative diseases. Alzheimer's researchers are particularly excited about recent developments in artificial intelligence (AI) and the possibility that these technologies will lead to even more discoveries. Notably, emerging AI technologies in Alzheimer's will fundamentally rely on the open‐source data collected by ADNI.

In addition to ADNI's open dataset, the overall ADNI initiative fostered an ecosystem of numerous other open and transparent bioresearch initiatives and created an incredibly rich environment for AI‐driven analysis that can propel the entire field of medicine forward.

## CONFLICT OF INTEREST STATEMENT

The authors declare no competing interests for this work. Author disclosures are available in the supporting information.

## Supporting information

Supporting Information

## References

[1] Weiner MW , Veitch DP , Aisen PS , et al. Impact of the Alzheimer's disease neuroimaging initiative, 2004 to 2014. Alzheimers Dement. 2015;11(7):865‐884. doi:10.1016/j.jalz.2015.04.005 26194320 PMC4659407

[2] Neurology Live . Clinical trial design in Alzheimer's disease research. 2024. Available at: https://www.neurologylive.com/view/clinical‐trial‐design‐in‐alzheimers‐disease‐research

[3] https://adni.loni.usc.edu/study‐design/#background‐container

[4] https://adni.loni.usc.edu/data‐samples/access‐data/

[5] Albala B , Appelmans E , Burress R , et al. The Alzheimer's disease neuroimaging initiative and the role and contributions of the private partners scientific board (PPSB). Alzheimers Dement. 2024;20(1):695‐708. doi:10.1002/alz.13483 37774088 PMC10843521

[6] McKhann GM , Knopman DS , Chertkow H , et al. The diagnosis of dementia due to Alzheimer's disease: recommendations from the national institute on aging – Alzheimer's association workgroups on diagnostic guidelines for Alzheimer's disease. Alzheimers Dement. 2011;7(3):263‐269. 21514250 10.1016/j.jalz.2011.03.005 PMC3312024

[7] van Dyck CH , Swanson CJ , Aisen P , et al. Lecanemab in early Alzheimer's disease. N Engl J Med. 2023;388(1):9‐21. 36449413 10.1056/NEJMoa2212948

[8] Budd Haeberlein S , Aisen PS , Barkhof F , et al. Two randomized phase 3 studies of aducanumab in early Alzheimer's disease. J Prev Alzheimers Dis. 2022;9(2):197‐210. 35542991 10.14283/jpad.2022.30

[9] Sims JR , Zimmer JA , Evans CD , et al. Donanemab in early symptomatic Alzheimer disease: the TRAILBLAZER‐ALZ 2 randomized clinical trial. JAMA. 2023;330(6):512‐527. 37459141 10.1001/jama.2023.13239 PMC10352931

[10] Jones‐Davis DM , Buckholtz N . The impact of the Alzheimer's disease neuroimaging initiative 2: what role do public‐private partnerships have in pushing the boundaries of clinical and basic science research on Alzheimer's disease? Alzheimers Dement. 2015;11(7):860‐864. 26194319 10.1016/j.jalz.2015.05.006 PMC4513361

[11] Chen C , Zissimopoulos JM . Racial and ethnic differences in trends in dementia prevalence and risk factors in the United States. Alzheimers Dement. 2018;4:510‐520. 10.1016/j.trci.2018.08.009 PMC6197734 30364652

[12] Alzheimer's Association . Causes and risk factors for Alzheimer's disease. https://www.alz.org/alzheimers‐dementia/what‐is‐alzheimers/causes‐and‐risk‐factors

